# Case report: Unraveling a unique case of male occult breast cancer with axillary intricacies and a comprehensive literature dive

**DOI:** 10.3389/fonc.2025.1374032

**Published:** 2025-02-11

**Authors:** Xintong Xie, Xiangyi Kong, Hongnan Jiang, Jidong Gao

**Affiliations:** ^1^ Department of Breast Surgical Oncology, National Cancer Center/National Clinical Research Center for Cancer/Cancer Hospital & Shenzhen Hospital, Chinese Academy of Medical Sciences and Peking Union Medical College, Shenzhen, China; ^2^ Department of Breast Surgical Oncology, National Cancer Center/National Clinical Research Center for Cancer/Cancer Hospital, Chinese Academy of Medical Sciences and Peking Union Medical College, Beijing, China

**Keywords:** male breast cancer, occult breast cancer, systemic therapy, anti-HER2 targeted therapy, surgery treatment

## Abstract

Male breast cancer is a rare neoplasm, accounting for approximately 1% of all breast cancer cases. It typically presents as a painless, retroareolar mass. An exceedingly rare variant is male occult breast cancer, which is primarily characterized by axillary lymph node enlargement without an identifiable primary breast tumor. We report an intriguing case of a septuagenarian patient diagnosed with male occult breast cancer. The patient presented with both axillary lymph node enlargement and an associated axillary skin ulcer, and was subsequently diagnosed with male occult breast cancer with metastases to the axillary and clavicular lymph nodes, as well as more distant sites. His treatment involved a multidisciplinary approach, including HER2-targeted therapy, chemotherapy, axillary lymph node dissection, and radiotherapy. Regular follow-ups have shown that his condition remains stable. Notably, this is the first documented case of male occult breast cancer with distant metastasis that was successfully treated with surgery and radiotherapy following systemic therapy. This case highlights the complex clinical presentation and management of male occult breast cancer. Our findings suggest that surgical intervention may be a feasible option post-downstaging by systemic therapy, even in the presence of distant metastases.

## 1 Introduction

Male breast cancer (MBC) is a rare disease, accounting for approximately 1% of all diagnosed breast cancer cases (1). Within this already limited subset, an even rarer variant is occult breast cancer (OBC), which typically presents as axillary lymph node metastasis without an identifiable primary tumor in the breast. OBC accounts for less than 1% of all breast cancer diagnoses. An exceptionally unusual subtype of OBC is male occult breast cancer (MOBC). A review of the literature reveals only eight previously reported cases of MOBC, with some ambiguity surrounding one of them, highlighting the extreme rarity of this condition.

In this case study, we report an elderly male patient who presented with axillary lymph node enlargement and an accompanying axillary skin ulcer during his initial hospital visit. This case is particularly significant as it represents the first documented instance of MOBC with multiple metastases, including distant spread. Following diagnosis, the patient was treated with a multidisciplinary approach, which included anti-HER2 targeted therapy and chemotherapy, leading to successful downstaging. Subsequently, the patient underwent surgery and radiotherapy, resulting in a stable disease state. The patient has remained in stable condition during follow-up.

This report aims to elucidate the clinical characteristics, diagnostic challenges, and management strategies for this exceedingly rare form of breast cancer. By doing so, we hope to contribute to the limited body of knowledge on MOBC and provide insights into the optimal treatment approach for patients presenting with advanced and metastatic disease.

## 2 Case report

### 2.1 Comprehensive diagnostic journey

**Figure 1:**
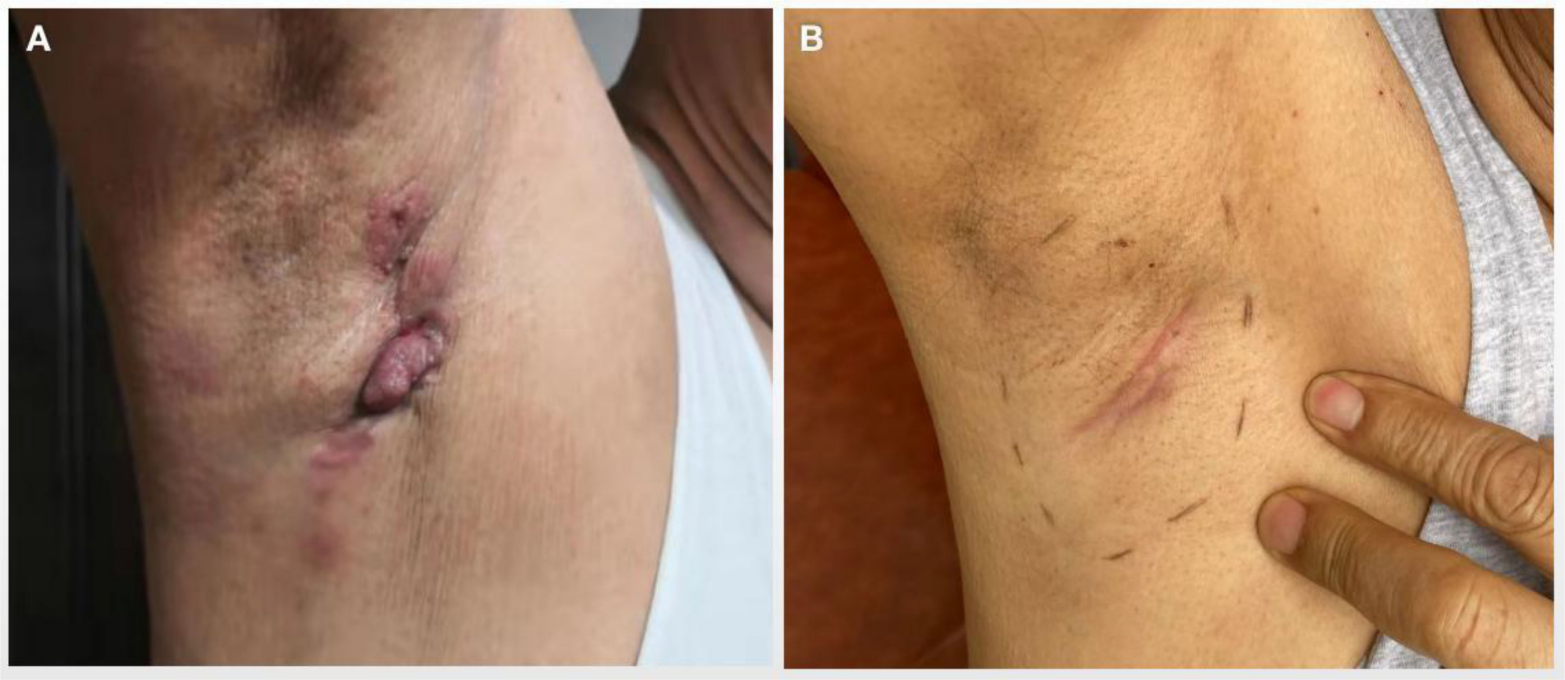
Skin nodules and ulcers in the right axilla of the patient. **(A)** Presentation of ulcerated axillary skin nodules at initial discovery. **(B)** Skin nodules disappeared significantly after several cycles of systematic treatment.

In 2019, a 71-year-old male presented to Xiangya Hospital of Central South University with a progressive, four-year history of right axillary lymph node swelling. More recent developments included axillary skin ulceration, further complicating his clinical presentation (**Figure 1A**). Upon evaluation, an immediate ultrasound revealed multiple solid nodules within the right axillary region. To better understand the nature and origin of these nodules, a histopathological examination of a needle biopsy from the right axillary lymph node was performed, confirming the presence of a poorly differentiated adenocarcinoma. The tumor’s characteristics strongly suggested a breast origin.

Immunohistochemical staining provided further diagnostic specificity, with the following results: CK-Pan(+), S-100(-), SOX10(-), GATA3(+), ER-, PR(30%+), HER2 3+, Ki-67 40%, TTF-1(-), NapsinA(-), CK7(+), CK20(-), Villin(-), CDX-2(-), CK5/6(-), 34βE12(+), AR(+), GCDFP-15(+), Mammaglobin(+), PSA(-), PSAP(-), PAX-8(-), S100P(+), P40(-). Following the diagnosis of malignancy, the patient underwent further diagnostic evaluations to assess the full extent of his condition. A repeat ultrasound confirmed the initial findings and also revealed metastatic involvement of the right supraclavicular fossa. Although the left supraclavicular lymph node was enlarged, no disease was detected in either breast.

A subsequent CT scan identified multiple metastatic lymph nodes in the right neck, bilateral supraclavicular regions, right axilla, right hilum, and mediastinum. Additionally, there was significant bone destruction in the left clavicle, necessitating further investigation to rule out metastasis. Other findings included increased density in the apical and anterior segments of the right upper lung and nodules in the right middle lung, suggestive of possible infectious lesions. A PET-CT provided more clarity, indicating a high likelihood of malignancy in the posterior and anterior segments of the right upper lobe. Furthermore, there was an abnormal metabolic increase in lymph nodes in the right hilum, mediastinum, right axilla, anterior chest wall, right clavicle region, and right neck IV-V region. This metabolic uptick was suggestive of metastatic tumors.

A gastroscopy revealed chronic superficial gastritis, but fortunately, a brain MRI showed no evidence of malignancy. Seeking a second opinion and potentially advanced treatment options, the patient visited Tianjin Cancer Hospital on December 18, 2019. Pathological consultation confirmed the initial diagnosis, with immunohistochemistry results aligning with those typical of breast cancer. Based on the accumulated diagnostic findings, the patient was conclusively diagnosed with MOBC, clinical stage IV (cTxN3M1), with extensive lymph node involvement and pulmonary metastasis.

The family history of the patient was not remarkable. The patient had no mutation in BRCA1/2 genes, and no other high-risk factors were found.

### 2.2 Comprehensive treatment journey and disease progression

Following his diagnosis, the patient began treatment at Xiangya Hospital, and later transitioned to the National Cancer Center/National Clinical Research Center for Cancer/Cancer Hospital Shenzhen Hospital. His initial treatment consisted of eight cycles of the PXH systemic therapy regimen, which included:

*Paclitaxel liposome: 175mg/m2 on Day 1*
*Capecitabine: 2500mg/m2 from Day 1 to Day 14*
*Trastuzumab: 8mg/kg in the first cycle and 6mg/kg in the subsequent cycle on Day 2*
*(Each cycle spanned three weeks.)*

After completing the eight cycles, physical examination revealed a significant reduction in the tumor, and the skin nodules had markedly regressed (**Figure 1B**). A CT scan in June 2020 confirmed partial response, with a decrease in the size of metastatic lymph nodes. However, these nodes remained adherent to the axillary vessels and surrounding muscles, making surgical intervention unfeasible. The CT scan also showed multiple mediastinal lymph node calcifications, though no malignant lesions were observed in the lungs. A breast MRI further confirmed the absence of lesions in both breasts.

During subsequent evaluations for potential surgery, the patient was administered:

*Trastuzumab: 6mg/kg on Day 1*
*Capecitabine: 2.0g in the morning and 1.5g in the afternoon from Day 1 to Day 14*

Following multidisciplinary consultations, the patient was started on a combined regimen of HP molecular targeted therapy and chemotherapy from July 2020 to October 2020, which included:

*HP Scheme:*
*Trastuzumab: 6mg/kg*
*Pertuzumab: 420mg, repeated every three weeks*
*Chemotherapy:*
*Capecitabine: 2.0g in the morning and 1.5g in the afternoon from Day 1 to Day 14, with a cycle every 21 days.*

**Figure 2:**
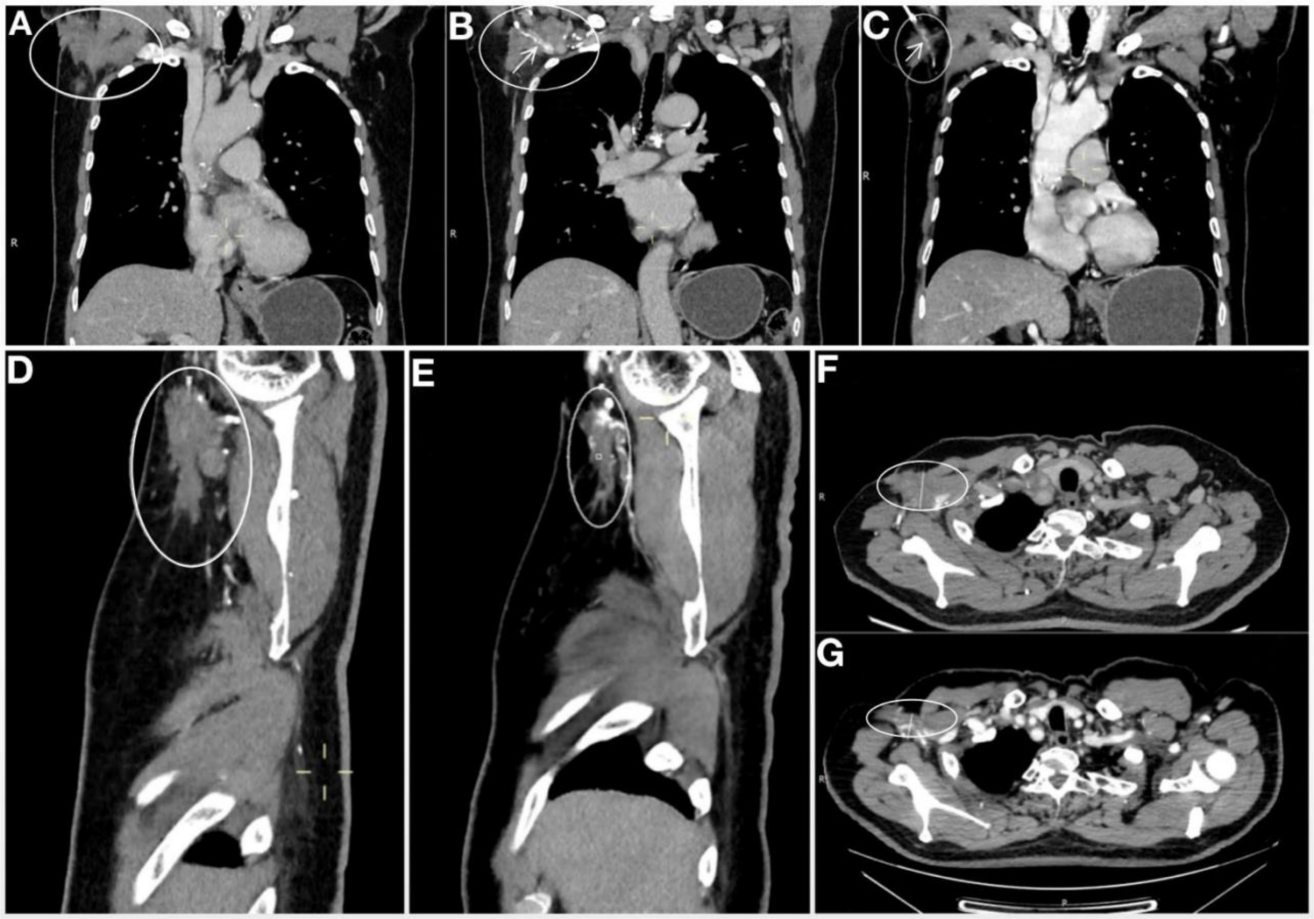
Comparison of right axillary CT after 2 cycle system treatment and before operation. **(A-C)** are the coronal plane imagine. **(A)** Imagine after 2 cycles of systematic treatment. **(B)** Imagine after 2 cycles of systematic treatment, demonstrating the mass tightly encircled the axillary vein. **(C)** Pre-operative image (Image after 8 cycles +6 cycles of systemic treatment), Compared with **Figures 3A, B**, the tumor was significantly reduced, and the tumor could be seen to surround part of the axillary vein. **(D)** Sagittal plane Imagine after 2 cycles of systematic treatment. **(E)** Sagittal plane Imagine pre-operative (after 8 cycles +6 cycles of systemic treatment). **(F)** Horizontal plane Imagine after 2 cycles of systematic treatment. **(G)** Horizontal plane Imagine pre-operative (after 8 cycles +6 cycles of systemic treatment). Imagine of sagittal plane and horizontal plane manifest that the tumor was significantly reduced before operation compared with post 2 cycles of systemic treatment, as well as coronal plane. The arrow marks the axillary vein, the oval marks the mass.

In November 2020, a re-evaluation for surgical viability showed promising results. The CT scan revealed further reduction in the lesion (**Figure 2**), now localized around the axillary vessels, with no other malignant formations detected. On November 4, 2020, an axillary lymph node dissection (ALND) was successfully performed under general anesthesia.

**Figure 3:**
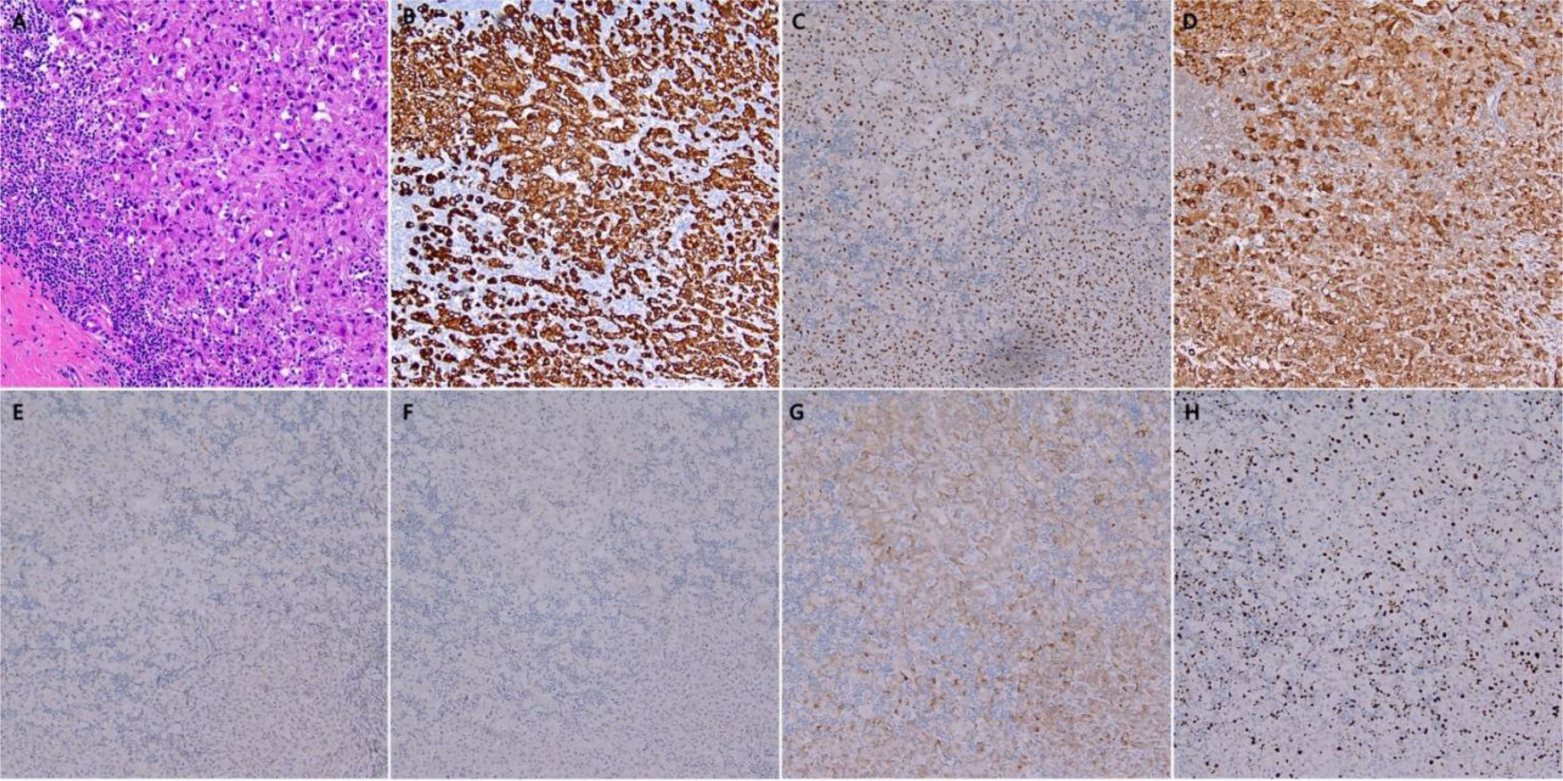
Histopathology of Surgical specimen. **(A)** Hematoxylin and eosin staining (magnification: ×20), showed moderate to severe regression post-treatment; **(B)** Immunohistochemistry for CK7; **(C)** Immunohistochemistry for GATA3; **(D)** Immunohistochemistry for GCDFP; **(E)** Immunohistochemistry for ER; **(F)** Immunohistochemistry for PR; **(G)** Immunohistochemistry for Her2; **(H)** Immunohistochemistry for Ki-67.

Post-operative pathological analysis revealed metastasis in 7 out of the 21 examined lymph nodes. Morphological and immunohistochemical studies showed apocrine differentiation, with some cells displaying a signet ring-like appearance, consistent with poorly differentiated adenocarcinoma, likely of mammary origin. Pathological review also indicated moderate to severe tumor regression following treatment (**Figure 3A**). While the tumor had breached the lymph node capsules and infiltrated the dermis, no invasion of the epidermis or skin appendages was observed. A series of immunohistochemical stains (**Figures 3B–H**) provided deeper insight into the tumor characteristics. The key findings were as follows:

1. *Identification of Primary Site:*
*AE1/AE3: 3+*
*CK7: 3+*
*GATA: 3+*
*GCDFP15: 3+*
*Mammaglobin: Negative*
*CK20: Negative*
*MUC5AC: Negative*
*TTF-1: Negative*
*CEA: 1+*
2. *Classification Identification.*
*CK5/6: Negative*
*EGFR: Negative*
*E-cad: Negative*
*P120: Positive in the tumor capsule (partial staining)*
3. *Breast Cancer Relevant Markers:*
*AR: Positive (> 90% in male cells)*
*ER: 0% (Negative)*
*PR: 0% (Negative)*
*HER2: 2+ (Further recommendation: FISH detection)*
*Ki-67: 30% Positive*
*P53: 10% weak (indicative of wild-type)*
*TOP2A: 20% Positive*
4. *Special Staining:*
*AB/PAS: Positive*
5. *FISH HER2:*
*No amplification detected.*

Following surgery, from November 2020 to March 2021, the patient underwent six cycles of dual-targeted therapy combined with capecitabine maintenance treatment, following a similar regimen to the one described earlier. From April to May 2021, the patient received comprehensive radiotherapy targeting the right breast and tumor bed. The specific radiotherapy protocol included:

*6MV-X-ray IMRT technology*
*Right chest wall + right axillary lymph node: 60Gy/2.4Gy/25f*
*Boost dose for right axillary lymph node region: 6Gy/2Gy/3f*
*Right supraclavicular lymph node drainage area: 50Gy/2Gy/25f*

In parallel, four cycles of targeted therapy were administered during and after radiotherapy, although these were discontinued prematurely due to the COVID-19 pandemic. The patient declined endocrine therapy. As of the latest follow-up in 2024, there has been no evidence of disease progression. For a detailed timeline of the patient’s disease course and treatments, refer to **Table 1**.

**Table 1:** Timeline of medical management and disease progression for the patient with MOBC.

Time	Medical management	Disease development
2016	None	Self-detected right axillary mass
2019.12	Diagnosed with MOBC, clinical stage IV.	Enlarging Mass; emerging ulceration.
2019.12- 2020.6	PXH*8 systemic therapy	PR
2020.6	Evaluating surgical opportunity. HX*1 systemic therapy	Surgery was still not feasible.
2020.7-2020.10	PHX*5 systemic therapy	PR
2020.11	ALND	NA
2020.11-2021.2	PHX*6 systemic therapy	NA
2021.3-2021.5	PH*4 systemic therapy	NA
2021.4-5	Radiotherapy.	NA
As of this case report		The disease has no progress and the patient was still alive

PXH, Paclitaxel liposome+ Capecitabine+ Trastuzumab Regimen; HX, Trastuzumab+ Capecitabine Regimen; PHX, Pertuzumab+ Trastuzumab+ Capecitabine Regimen; PH, Pertuzumab+ Trastuzuma Regiment; ALND, axillary lymph node dissection; PR, partially response; NA, not applicable.

## 3 Discussion

MBC is a rare disease, accounting for approximately 1% of all breast cancer cases (1). Within this already small subset, OBC is an even more uncommon presentation, representing 0.3-1% of breast cancer diagnoses (2, 3). OBC is typically characterized by axillary lymph node metastasis without an identifiable primary tumor in the breast, making it a challenging entity to diagnose. The combination of male gender and occult presentation further narrows the incidence, with only a handful of MOBC cases reported in the literature. A comprehensive review of the literature identified eight reported cases of MOBC, which are systematically summarized in **Supplementary Table S1**. The patients ranged in age from 29 to 84 years, and most were diagnosed at an advanced stage of disease. Seven of the eight cases underwent surgical intervention, with varying degrees of success.

In this report, we present a unique case of MOBC, notable for being the first documented instance of MOBC with distant metastasis. This patient exhibited metastasis to multiple sites, including the axillary and supraclavicular lymph nodes, as well as pulmonary involvement, adding complexity to the diagnostic and therapeutic approach. Despite the advanced stage at presentation, the patient responded favorably to a multidimensional treatment strategy, including anti-HER2 molecular targeted therapy, chemotherapy, surgical intervention, and radiotherapy, leading to a stable disease state. The objective of this discussion is to underscore the clinical complexities of MOBC, the diagnostic challenges it presents due to the absence of a primary tumor, and the effectiveness of a comprehensive, multidisciplinary treatment approach in managing this rare and advanced form of breast cancer.

### 3.1 Diagnostic challenges

Diagnosing MOBC presents a unique set of challenges, primarily due to the absence of a detectable primary breast tumor. In typical cases of breast cancer, the presence of a palpable mass guides clinicians toward diagnostic imaging and biopsy of the breast. However, in MOBC, the initial presentation is often limited to axillary lymph node metastasis, which can easily be mistaken for other malignancies, such as lymphoma, melanoma, or adenocarcinomas of gastrointestinal or pulmonary origin. This diagnostic ambiguity often leads to delays in establishing the correct diagnosis.

The standard diagnostic workup for MOBC includes a combination of imaging modalities and biopsy. Bilateral mammography and breast ultrasound are typically performed to identify any hidden breast lesions, though these modalities often fail to detect a primary tumor in MOBC. Magnetic resonance imaging (MRI) may provide additional clarity but can also be inconclusive (4). Ultimately, a definitive diagnosis relies on histopathological and immunohistochemical analysis of the axillary lymph node biopsy.

Pathologically, microscopic evaluation is pivotal in discerning whether a tumor bears characteristics of an adenocarcinoma, squamous carcinoma, or lymphoma. Tumor nests boasting sieve-like or comedo-like structures robustly corroborate an origin from breast cancer. In contrast, adenocarcinomas exhibiting tall columnar cells secreting mucus predominantly herald from the stomach or the large intestine. The intracellular presence of melanin granules lends weight to a diagnosis of melanoma.

Immunohistochemical staining plays an indispensable role in pinpointing the primary source of axillary metastatic lymph nodes. Positive results for GCDFP, Mammaglobin, and GATA3 are compelling indicators suggesting the lesion’s genesis in the mammary gland (5). Markers such as S-100, Sox-10, HMB-45, MITF, and MART-1 align with malignant melanoma. TTF-1 and NapsinA manifest positivity in cases of lung adenocarcinoma. Gastrointestinal adenocarcinomas frequently present as CK7-positive and CK20-positive (5). In the given scenario, the immunohistochemical palette rendered results such as GCDFP-15(+), Mammaglobin(+), GATA3(+), S-100(-), SOX10(-), TTF-1(-), NapsinA(-), CK7(+) and CK20(-). Post pathological consultation, the diagnosis was crystallized as breast cancer.

In our case, the diagnostic process was further complicated by the presence of distant metastases and the patient’s prolonged history of untreated axillary lymph node swelling. Despite multiple imaging studies, including mammography, ultrasound, and MRI, no primary tumor was identified in the breast, leading to reliance on immunohistochemical staining to confirm the diagnosis of breast cancer. The diagnostic delay, exacerbated by the atypical presentation and absence of a breast lesion, underscores the difficulties clinicians face in promptly identifying MOBC.

### 3.2 Therapeutic approaches and rationale

MOBC with distant metastasis requires a multidisciplinary approach, combining systemic therapy, surgery, and radiotherapy to achieve optimal outcomes. The patient’s perspective was to prolong survival while avoiding treatment with severe side effects or unnecessary damage from the treatment. This case report accentuates the paramount importance of tailoring systemic treatment plans based on clinical assessments, current scientific literature and individual patient’s perspectives. In addition to this, the patient was performed local treatment with minimal damage. As result, multifaceted treatment achieved a favorable prognosis and was tolerated by the patient in this case.

The following sections will outline the rationale behind each component of the treatment plan, including the use of systemic therapy to downstage the tumor, surgical intervention for local control, and radiotherapy to target residual disease and prevent recurrence.

1. *Systemic Therapy:* The initial systemic therapy regimen in our patient consisted of PXH (paclitaxel, capecitabine, and trastuzumab), a combination designed to target the aggressive nature of HER2-positive breast cancer. The CHAT study verified that for patients tolerant to dual chemotherapy, combining trastuzumab with docetaxel and capecitabine offers superior outcomes compared to pairing trastuzumab with docetaxel alone (6). Docetaxel and paclitaxel are the same type of drugs Paclitaxel and capecitabine were selected for their proven efficacy in controlling metastatic breast cancer, while trastuzumab, a monoclonal antibody targeting the HER2 receptor, was included to directly inhibit the HER2-positive tumor cells. This regimen played a critical role in downstaging the tumor, as evidenced by the significant reduction in metastatic lymph node size and the disappearance of skin nodules post-therapy. The use of trastuzumab and pertuzumab in HER2-positive breast cancer is well-supported by the CLEOPATRA study, which demonstrated that dual-targeted therapy with trastuzumab and pertuzumab, in combination with chemotherapy, significantly improved progression-free survival (PFS) and overall survival (OS) in patients with HER2-positive breast cancer (7). In our case, financial constraints initially prevented the use of pertuzumab. However, after eight cycles of PXH, the treatment strategy shifted to the HP (trastuzumab + pertuzumab) regimen, along with capecitabine, aligning with evidence from studies like CLEOPATRA trial. This regimen further downstaged the tumor.
2. *Surgical Intervention and Radiotherapy*: The decision to proceed with axillary lymph node dissection (ALND) was made following significant downstaging of the tumor after systemic therapy. The therapeutic efficacy of primary tumor excision on the survival of stage IV breast cancer patients remains a topic of debate. Of the four randomized controlled trials published to date, three have not evidenced an elevated survival rate post-local excision in stage IV breast cancer patients (8–11). Notably, the MF07-01 randomized controlled trial (RCT) (10) revealed a 14% higher likelihood of OS among newly diagnosed stage IV BC patients who underwent local surgical treatment combined with chemotherapy compared to those who only received chemotherapy. A meta-analysis incorporated data from 714 patients across three RCTs and 67,272 patients from 30 observational studies. The findings substantiate that local surgical intervention significantly bolsters OS, with subgroup analysis suggesting benefits predominantly among patients with a single metastatic site, bone-only metastasis, and those achieving negative surgical margins (12). Assessing individual patient scenarios, if no distant metastatic lesions are observed post-systemic treatment, surgical intervention might promise a better prognosis and might potentially enhance the patient’s quality of life by alleviating axillary skin affliction.

For OBC patients with axillary lymph node metastasis, axillary lymph node dissection stands as the contemporary standard surgical approach. Given the high recurrence risk in the axillary region for patients with numerous affected axillary lymph nodes or those with irregular, fixed axillary lymph nodes after surgical intervention alone, most OBC patients should undergo axillary radiotherapy (13).

Contemporary debate on the local treatment of OBC has largely concentrated on breast intervention strategies. Since the Halsted era, the classic surgical intervention for OBC has been the removal of the ipsilateral breast and axillary lymph node dissection. A survey conducted in 2005 by the American Association of Breast Surgeons, revealed a divided preference: 43% endorsed mastectomy for breast cancer, and 37% advocated for whole breast radiation (14). Research has also posited that whole breast radiation as an alternative to mastectomy is feasible, albeit necessitating standardized radical breast irradiation. Ellerbroek’s study discerned no significant disparities in long-term survival rates between radiation and mastectomy patients (15).

In our case, the patient’s preoperative imaging did not detect primary breast lesions. After axillary lymph node dissection, he underwent right whole breast radiation. Given the initial diagnosis of axillary metastasis, suspected distant metastasis (as per radiological evaluation), which receded post systemic treatment, the patient was also subjected to radiation of the right axilla and supraclavicular lymph node drainage area, resulting in a favorable prognosis.

### 3.3 Limitations and areas for improvement

While this case illustrates a successful treatment outcome for MOBC with distant metastasis, several limitations warrant discussion.

First, little data was available when axillary lymph nodes swollen were firstly identified in 2016. It hinders a more comprehensive understanding of the disease’s progression prior to diagnosis. The patient experienced axillary lymph node swelling for an extended period before seeking medical attention, which likely delayed the diagnosis and allowed the disease to progress to an advanced stage. Earlier detection may have permitted a less aggressive disease course, potentially reducing the extent of metastasis and improving long-term prognosis. Second, the patient declined endocrine therapy, which is typically recommended for hormone receptor-positive breast cancer. Although the patient received effective HER2-targeted therapy, the absence of endocrine treatment may influence long-term disease control and increase the risk of recurrence. Endocrine therapy plays a crucial role in reducing the likelihood of disease relapse, and its omission represents a limitation in the overall treatment strategy. Finally, the COVID-19 pandemic disrupted the patient’s targeted therapy regimen, as four cycles of post-radiotherapy targeted therapy were prematurely halted. The interruption in treatment raises concerns about whether the full benefit of the dual-targeted therapy (trastuzumab and pertuzumab) was realized. Continuation of this regimen might have further enhanced the patient’s disease control and long-term prognosis. Uninterrupted treatment could have potentially led to an even more robust therapeutic response.

## 4 Conclusion

This is the first case of a MOBC patients with distant metastases that has undergone surgical intervention post-downstaging by systemic therapy and recent regular follow-ups in 2024 have shown that his condition has remained stable. This case underscores the difficulty and extreme importance of early detection of MOBC, which was the biggest lesson from this case. It suggests dual-targeted therapy (trastuzumab and pertuzumab) played a pivotal role in downstaging the tumor, enabling surgical resection, and contributing to long-term disease control for HER2-positive disease. It also emphasizes that surgical intervention can be a viable option even for distant metastasized MOBC patients those who post-downstaging after systemic therapy.

Future research should focus on optimizing treatment protocols, particularly exploring the long-term benefits of targeted therapies in MOBC, to further improve survival and quality of life in this rare and challenging disease.

## Data Availability

The original contributions presented in the study are included in the article/**Supplementary Material**. Further inquiries can be directed to the corresponding authors.
