# Supplementary material for: Case report: Unraveling a unique case of male occult breast cancer with axillary intricacies and a comprehensive literature dive
Source: Front Oncol. 2025 Feb 11;15:1374032. doi: 10.3389/fonc.2025.1374032 (PMC11851083; doi:10.3389/fonc.2025.1374032)
Supplement: Supplementary file 1 [file Table1.docx]

| First author | Published year | Age | Symptom | Tumor distribution | Subtype | Stage | Treatment | | | Prognosis |
| --- | --- | --- | --- | --- | --- | --- | --- | --- | --- | --- |
|  |  |  |  |  |  |  | Surgery | Radiotherapy | Neoadjuvant/ adjuvant/ systemic therapy |  |
| Mengna He | 2015 | 40 | Palpable masses | Left axilla | Her2+ | IIIC | ALND +mastectomy | Y | Neoadjuvant(not finished)+chemotherapy +endocrine therapy | Lung metastasis |
| Xinyu Wang | 2018 | 49 | Painless mass | Left axilla | Luminal(HER2-) | IIIC | ALND+ infraclavicular lymph node dissection | Y | Chemotherapy +endocrine therapy | no signs of recurrence or metastasis. |
| Guo-Li Gu | 2009 | 72 | Painless and enlarged mass | Right axilla | Her2+ | IIA | ALND +mastectomy | N | / | no signs of recurrence or metastasis. |
| Xin-Hua Wang | 2022 | 64 | Enlarging, painful mass | Right axilla | TNBC | IIIC | / | N | Neoadjuvant(not finished)+chemotherapy +endocrine therapy | PD ,then SD |
| Ruixin Xu | 2017 | 29 | Painless nodule | Left axilla | Her2+ | IIIC | ALND | Y | Chemotherapy | no signs of recurrence or metastasis. |
| Le Zhang | 2017 | 84 | Palpable nodule | Right axilla | TNBC | IIA | ALND +mastectomy | N | Chemotherapy | no signs. |
| Sung Mo Hur | 2012 | 59 | Palpable mass | Right axillary | Luminal(HER2-) | IIA | ALND +breast skin-sparing mastectomy | N | Chemotherapy +endocrine therapy | no signs of recurrence or metastasis. |
| Sung Mo Hur | 2012 | 45 | Palpable lesion | Left axilla | Luminal(HER2-) | IIIA | ALND | Y | Chemotherapy +endocrine therapy | no signs of recurrence or metastasis. |
| Hiroshi Takeyama* | 2010 | 58 | Mass | Right axillary | Luminal(HER2-) | / | ALND | N | Endocrine therapy | Not mention |

**Supplementary materials, table S1** Case reports of MOBC of the breast. *This case was unclear weather it’s MOBC or accessory mammary gland cancer. ALND, axillary lymph node dissection; Y, yes; N, no; PD, progressive disease; SD, stable disease
